# Trends in tobacco sales in Germany

**DOI:** 10.17886/RKI-GBE-2017-046

**Published:** 2017-06-14

**Authors:** Benjamin Kuntz, Johannes Zeiher, Cornelia Lange, Thomas Lampert

**Affiliations:** Robert Koch Institute, Department for Epidemiology and Health Monitoring, Berlin, Germany

**Keywords:** TOBACCO, CIGARETTES, SMOKING, TRENDS, TOBACCO TAX REVENUE STATISTICS

## Abstract

Based on data from the tobacco tax revenue statistics provided by Germany’s Federal Statistical Office, we analyse the development of sales of various tobacco products. In 2016, per capita consumption of tobacco products taxed in Germany was 918 manufactured cigarettes, 37 cigars/cigarillos, 308 g fine cut (equivalent to 462 cigarettes), and 31 g of (water) pipe tobacco. Between 1991 and 2016, the sale of manufactured cigarettes decreased by nearly half, while the sale of fine cut increased by around two thirds. If the amount of fine cut is expressed as cigarette equivalents (whereby 1kg of fine cut equals 1,500 cigarettes) and added to the number of manufactured cigarettes sold, then cigarette sales have decreased by one third since 1991. Numerous facts indicate that measures implemented in the context of a more restrictive tobacco control policy, such as tax increases and smoking bans, have contributed to this decrease in tobacco sales.

## Introduction

Smoking is a health hazard that increases the risk of contracting severe diseases and dying prematurely [1, 2]. Reducing the population’s consumption of tobacco therefore remains a fundamental health policy goal [3, 4]. The planning and evaluation of tobacco prevention and control policy measures requires meaningful, regularly gathered data on the spread of tobacco use in the population [3, 5]. Conclusions about the spread of tobacco consumption usually depend on representative population surveys (see the Smoking among adults in Germany fact sheet based on data from GEDA 2014/15-EHIS in this issue of the Journal of Health Monitoring). The available surveys show that the share of smokers in Germany has decreased over the past years, a fact that holds particularly true for adolescents and young adults, with a more marked decrease among men than women [5-10].

Along with population surveys, data from the statistics on tobacco tax revenue from Germany’s Federal Statistical Office helps estimate tobacco consumption. This data provides information on sales and the prices of tobacco products, as well consumer expenditure and tax revenue. After energy, the levy on tobacco is Germany’s most important excise. In 2016, Germany raised EUR 14.1 billion in tobacco tax revenue [11]. Beyond producing revenue, however, tobacco also results in high costs to the economy. The direct annual healthcare costs for treating tobacco-related diseases and health problems amount to an estimated EUR 25.4 billion. To this we must add the indirect costs of people unable to work due to sickness, early retirement, and premature death. Estimates therefore reckon with total annual costs to the economy of around EUR 79.1 billion [1, 12].

## Indicator

The data provided in the following comes from the statistics of the German Federal Statistical Office on tobacco tax revenue [11, 13, 14]. Germany’s tobacco taxation law defines the content and form of this data. Data on tobacco tax revenue is collected based on the tax declarations (ordered and returned excise stamps) of companies that produce or import tobacco products. The central excise stamp agency in the city of Bünde prepares the data from tax declarations and transmits it to the Federal Statistical Office for analysis and general publication.

**Figure 1:**
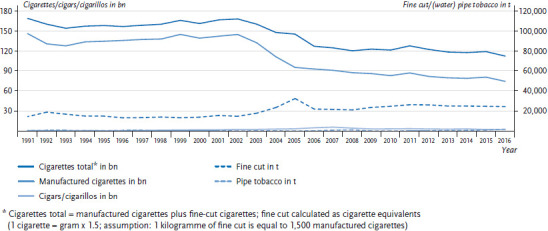
Absolute number of taxed tobacco products in Germany (1991 to 2016) Source: Tobacco tax revenue statistics [11, 13, 14]

Statistics on tobacco tax revenue provide quarterly and annual data on the number of (1) manufactured cigarettes, (2) cigars and cigarillos, (3) fine cut, and (4) pipe tobacco (including water pipe tobacco) taxed in Germany. In the following we provide an analysis of the development of sales for each of these four products in Germany from 1991 to 2016. Figures for the number of cigarettes, cigars, and cigarillos sold are given in billions, for fine cut and pipe tobacco in tonnes (Figure 1). Moreover, figures for cigarettes, cigars, and cigarillos are also given as units per person, and for fine cut and pipe tobacco as grams per person (per capita consumption, Table 1). To calculate total cigarette consumption, one kilogramme of fine cut is considered equivalent to 1,500 manufactured cigarettes. Fine cut consumption is subsequently converted into an equivalent amount of cigarettes and this figure is then included in the cigarette total [15, 16].

## Results and discussion

According to the statistics on tobacco tax revenue, around 112.8 billion cigarettes were sold in Germany in 2016. This figure includes 75.0 billion manufactured cigarettes and 25,188 tonnes of fine cut (Figure 1) [11]. Per capita, this translates into an average of 918 cigarettes and 462 cigarettes made from fine cut (308 g of fine cut per capita, Table 1). Cigars and cigarillos accounted for 3.0 billion units (37 per capita). Moreover, 2,521 tonnes of pipe tobacco were sold (31 g per capita, Figure 1, Table 1).

Between 1991 and 2002, sales of manufactured cigarettes and fine cut, the most important tobacco products with regard to the amounts consumed, remained relatively stable (Figure 1). Following the massive increase in levies on tobacco, which led to a significant increase in the price of cigarettes between 2002 and 2005, cigarette consumption dropped by around one third within three years, from 145.2 to 95.8 billion cigarettes (-34.0%). Simultaneously, sales of fine cut, to which a lower tax rate applied, more than doubled, rising from 15,473 to 33,232 tonnes (+114.8%). In 2006, when Germany adjusted the levies applied to fine cut, sales initially collapsed, then recovered between 2008 and 2011 and have remained relatively stable since. Manufactured cigarette sales did not recover after their collapse and instead continued to decrease, albeit more slowly than between 2002 and 2005.

During the phase between 1991 and 2016, the total amount of taxed cigarettes in Germany decreased by one third, from 169.2 to 112.8 billion. This figure includes manufactured cigarette sales, which were nearly cut in half, dropping from 146.5 to 75.0 billion (Figure 1). Sales of fine cut increased by nearly two thirds during this time. In spite of this absolute increase in the amount of fine cut sold, per capita consumption of cigarettes dropped from 2,116 to 1,380 (-736 units or -34.8%, Table 1).

As a share of the total amount of tobacco sold, the consumption of cigars, cigarillos, and pipe tobacco is negligible. Only 1.2% (EUR 168 million) of net tobacco tax revenue resulted from the sale of cigars, cigarillos, and pipe tobacco [5, 11]. During the period analysed, consumption of these tobacco products was subject to fluctuation, yet it increased overall (Table 1). Over the past years, the increased popularity of water pipes (also known as shisha or hookah) among adolescents and young adults in Germany has probably led to the increased demand for special water pipe tobacco [1, 17, 18].

Since the data from the statistics on tobacco tax revenue counts only the tobacco products taxed in Germany, actual tobacco consumption is probably higher than these figures suggest. These statistics do not include tobacco products imported illegally (or legally) into Germany but taxed outside of Germany. The ratio of tobacco products that are not taxed in Germany to total tobacco sales according to tobacco tax revenue statistics is largely unknown. A so-called ‘discarded cigarette pack’ study by the tobacco industry estimates that one in five to one in six cigarettes smoked in Germany are not taxed in the country [19]. For 2016, an estimated 18% of cigarettes were not taxed in Germany, with great differences between western and eastern Germany (12.2% compared to 39.5%) [19]. How reliable these estimates are and how high the percentage of smuggled cigarettes among the cigarettes not taxed in Germany is remains controversial. The survey methodology has been criticised, with many doubting the survey’s representativeness [20, 21]. Overall, tobacco tax revenue data points to a significant decrease in cigarette sales in Germany over the past 25 years. Considering the declining prevalence of smoking in the population, such a trend seems plausible [5, 6, 8]. The degree to which the spread of electronic inhalers, the most prominent of which is the electronic cigarette, has contributed to decreasing tobacco cigarette sales is unclear. But even though the effect of individual measures is hard to quantify, tobacco prevention measures and control policies have, since the early 2000s, certainly contributed significantly to this development [1, 3, 5]. In particular, a pronounced decrease in cigarette sales accompanied the sharp increase in the tax levied on tobacco products between 2002 and 2005. Even though some smokers ended up switching to fine cut and rolling their own cigarettes because of the lower tax rate that applied to fine cut, total cigarettes sales still dropped. Further important measures to reduce smoking included legislation to protect non-smokers from secondhand smoke in the workplace (2002), the ban on selling tobacco products to minors under 18 (2007), and federal and state legislation for the protection of non-smokers (after 2007). Finally, since the revision of the EU’s Tobacco Products Directive, implemented by Germany in 2016, at least two thirds of the front and back surface of cigarette packs need to be printed with pictures and warnings, i.e. a combination of written warnings and what are called shocking images in Germany that highlight the health consequences of smoking [5]. German tobacco control policy, notwithstanding the measures already implemented, is considered to be only tentative, at least in international comparison [22]. Within this context, the recommendations made by important stakeholders to reduce demand for tobacco products need to be discussed further and considered as options for future action. The German Cancer Research Center [1] and the German Alliance against Non-communicable Diseases [23] recommends further tax increases and measures such as a ban on tobacco advertisements in public spaces, the elimination of exceptions to smoking bans at the federal state level (in gastronomy, for example), and the expansion of tobacco cessation programmes.

## Key statements

- In 2016, around 112.8 billion cigarettes (including fine-cut tobacco) were sold in Germany, roughly 1,380 cigarettes per capita.
- Over the past 25 years, the total amount of taxed cigarettes in Germany has dropped by around one third.
- Increasing taxes on tobacco led to a 34% cut in cigarette sales between 2002 and 2005 alone.
- The German population today smokes 736 cigarettes fewer per capita than in 1991.
- Cigars, cigarillos, and pipe tobacco remain a niche market, while sales of water pipe tobacco have increased over the past years.

## Figures and Tables

**Table 1:** Taxed tobacco products in Germany per capita (1991 to 2016) Source: Tobacco tax revenue statistics [11, 13, 14], current population estimate*

Year	Manufactured cigarettes	Cigars/cigarillos	Fine cut (cigarettes)	Pipe tobacco
	Per capita	Per capita	Grams (units**) per capita	Grams per capita
1991	1,831	17	190(285)	16
1992	1,627	16	243(365)	15
1993	1,578	14	149(224)	15
1994	1,646	17	139(209)	14
1995	1,654	13	137(206)	13
1996	1,664	17	136(204)	13
1997	1,678	19	142(213)	13
1998	1,687	24	148(222)	12
1999	1,770	28	154(231)	12
2000	1,699	31	155(233)	11
2001	1,731	31	168(252)	11
2002	1,760	37	188(282)	10
2003	1,607	38	225(338)	11
2004	1,355	44	294(441)	11
2005	1,162	49	403(605)	10
2006	1,135	67	276(414)	11
2007	1,112	79	272(408)	20
2008	1,071	61	266(399)	23
2009	1,058	46	298(447)	10
2010	1,022	49	312(468)	9
2011	1,092	53	337(506)	11
2012	1,025	47	335(503)	13
2013	995	44	319(479)	15
2014	982	48	317(476)	17
2015	995	36	312(468)	21
2016***	918	37	308(462)	31

* Current population estimates: 1991-2010 based on earlier census data; 2011-2016: based on the 2011 census

** Fine cut expressed as cigarette equivalents (1 cigarette = gram x 1.5; assumption: 1 kilogramme of fine cut is equivalent to 1,500 manufactured cigarettes)

*** Preliminary results
